# A Comparative Study on the Effect of Different Methods of Recycling Orthodontic Brackets on Shear Bond Strength

**DOI:** 10.1155/2021/8844085

**Published:** 2021-01-21

**Authors:** Purna Prasad Khanal, Basanta Kumar Shrestha, Rajiv Yadav, Sanjay Prasad Gupta

**Affiliations:** ^1^Pokhara Academy of Health Sciences, Pokhara, Nepal; ^2^Chitwan Medical College, Chitwan, Bharatpur, Nepal; ^3^Institute of Medicine, Maharajgunj, Kathmandu, Nepal; ^4^Tribhuvan University Dental Teaching Hospital, Institute of Medicine, Maharajgunj, Kathmandu, Nepal

## Abstract

**Aim:**

The aim of this study was to evaluate and compare the effect of different methods of recycling stainless steel orthodontic brackets on shear bond strength.

**Methods:**

One hundred twenty human premolars extracted for orthodontic purpose were randomly divided into four groups. Standard MBT (0.022″) brackets were bonded on the buccal surface of all samples with light cured adhesive primers using an LED curing unit for 10 seconds. Group I was assigned as control, and the brackets of Group II, Group III, and Group IV were subjected to recycling by flaming, flaming with sandblasting, and flaming with ultrasonic cleaning, respectively. The recycled brackets were rebonded, and final debonding of all brackets was performed using a universal testing machine at a crosshead speed of 0.5 mm/min and shear bond strength was determined. Data were analyzed with descriptive statistics, ANOVA, and post hoc tests. The adhesive remnant index was evaluated using a stereomicroscope at 10X magnification.

**Results:**

The highest shear bond strength was obtained with Group I (10.35 ± 0.46 MPa), followed by Group III (9.36 ± 0.55 MPa) and Group IV (5.97 ± 0.66 MPa), and the least value was obtained with Group II (4.30 ± 0.55 Mpa). Significant differences among the groups were detected by analysis of variance. Tukey's post hoc multiple comparison test showed that the shear bond strength of each group was significantly different from one another (*p* < 0.001).

**Conclusions:**

Shear bond strength of new brackets was significantly higher than that of the recycled brackets. Among recycled brackets, flaming with sandblasting provided adequate shear bond strength, flaming with ultrasonic cleaning provided a borderline value for clinical use, and flaming alone led to a significantly lower value.

## 1. Introduction

Bonding of brackets on tooth surface is a principal requirement in contemporary fixed orthodontic treatment. With the introduction of enamel etching by Buonocore and direct bonding system by Newman, bonding of brackets became relatively more convenient. Nowadays, preadjusted brackets are more popular, which bear inbuilt features to compensate for the first, second, and third order bends [1]. These inventories increase the cost of the brackets, so replacing the debonded or old brackets with a new one makes the orthodontic treatment more expensive. One possible alternative to the replacement with new bracket is to recycle the old or debonded bracket and rebond on tooth surface. The major advantage of recycling is the economic saving, which could be as high as 90 percent, due to the fact that a single bracket can be reused up to five times [2]. Commonly used recycling methods include roughening of debonded attachment with greenstone, direct flaming, sandblasting, use of chemical solvents, ultrasonic cleaning etc. [3–5].

In the literature, there are not clear guidelines about shear force limits, but in fact a good biomaterial should allow good adhesion in order to sustain masticatory forces, but bonding values should not be too strong in order to avoid substrate loss. Therefore, the ideal biomaterial should have bonding forces included in the interval of 5–50 MPa, even if these limits are mostly theoretical [6]. Brackets should not be adversely affected after recycling with different methods. Previous studies have reported that recycling with flaming results in shear bond strength below the recommended range of clinical need [4, 5, 7, 8], while recycling with sandblasting gives clinically acceptable shear bond strength [4, 8–13]. SBS of brackets recycled by flaming with sandblasting was reported much less in a study by Gupta et al. (2.05 MPa) [10], and the large value (26.94 MPa) was reported in a study by Bansal and Bansal [11]. However, limited studies are available in literature about the effects of recycling orthodontic brackets with ultrasonic cleaning on shear bond strength. Quick et al. [5] and Kumar et al. [14] reported shear bond strength of brackets recycled with ultrasonic cleaning less than the recommended bond strength (less than 6 MPa), while Chetan [4] reported this within the recommended range. Hence, this study aimed to evaluate and compare the effect of different methods of recycling stainless steel orthodontic brackets on shear bond strength while rebonding. Working null hypothesis was set as there is no difference in shear bond strength of stainless steel orthodontic brackets recycled with different methods.

## 2. Materials and Methods

The study was an in vitro experimental study conducted at Orthodontics & Dentofacial Orthopedics Unit, Department of Dentistry, Tribhuvan University Teaching Hospital, Maharajgunj Medical Campus, Maharajgunj, Kathmandu, Nepal, in coordination with the Nepal Bureau of Standards and Metrology, Balaju, and Nepal Agricultural Research Council (NARC), Khumaltar. Ethical clearance was obtained from the Institutional Review Board. This study considered (95% CI) and 90% power to estimate the sample size based on a similar type of previous research [4]. For this purpose, the mean ± SD value of the intervention group 7.4463 ± 0.8870 and the mean ± SD value of the control group 8.4460 ± 2.2108, respectively, were taken. Using the formula, Sample Size (*n*)=((2*σ*^2^((*zα*/2)+(*zβ*/2)^2^)/(*μ*_1_ − *μ*_2_)^2^), it was calculated as 105, which consisted 26.25 samples on 4 different groups. As a round figure, we selected 30 samples in each group altogether comprising 120. One hundred twenty human first premolars extracted for orthodontic treatment purpose were used in this study, and nonprobability convenience sampling technique was applied.

Inclusion criteria were human premolars with extraction time less than 4 months, intact buccal surface, and immersed in distilled water as a storage solution (15, 16), while the exclusion criteria were those with developmental defects, cracks caused by the extraction forceps, dental caries, and the teeth subjected to any pretreatment chemical agent.

Custom fabricated moulds were used to make acrylic blocks (Rapid Repair; Dentsply India Pvt. Ltd, Delhi, India), and the teeth thus collected were mounted on an acrylic block such that the roots were completely embedded into the acrylic up to the cementoenamel junction leaving the crown exposed. The labial surfaces of the teeth were kept perpendicular to the bottom surface of the mould [15]. Each sample was assigned number 1 to 120 and randomly divided into 4 groups.

- Group I: control (new brackets, assigned with “C”)
- Group II: flaming group (assigned with “F”)
- Group III: flaming with sandblasting group (assigned with “S”)
- Group IV: flaming with ultrasonic cleaning group (assigned with “U”)

Before bonding, the buccal surfaces of the teeth were cleaned with fine pumice powder (DPI, New Delhi, India) in water using a cup [4, 5, 8]. The buccal surface of each tooth was etched for 30 seconds with 37% phosphoric acid gel (Ormco corp., Orange, CA, USA) [16–18]. Each tooth was then rinsed with a distilled water spray for 5 seconds and dried with oil-free air till the etched tooth will appear chalky white [11, 14, 15, 19]. A thin coat of light cure adhesive primer, Orthosol (Enlight; Ormco corp., Orange, CA, USA), was applied to the acid-etched enamel. Light cure adhesive resin (Enlight; Ormco corp., Orange, CA, USA) was applied on the 0.022″ slot MBT stainless steel double mesh premolar bracket base (Leone, Sesto Fiorentino, Italy) having a surface area of 11.6 mm^2^ (provided by the manufacturer), which was then placed on the teeth with a reverse tweezers near the centre of the buccal surfaces [15]. Light curing was performed using Rainbow LED curing light (Qingdao Hungyun Trade Co., Ltd, Shandong, China) for 10 seconds [11]. The light intensity measured using a radiometer (CM300-2000; APOZA, New Taipei City, Taiwan) was 830 mW/cm^2^. Group II, Group III, and Group IV brackets were subjected to recycling, and Group I brackets were stored in distilled water until final debonding using a universal testing machine to measure shear bond strength.

**Figure 1:**
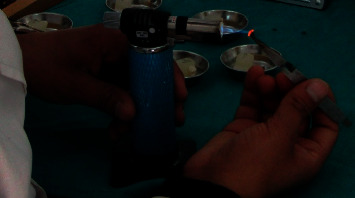
Flaming of brackets.

**Figure 2:**
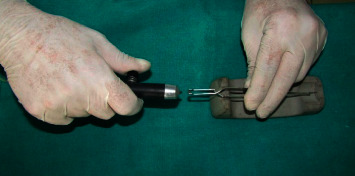
Sandblasting of brackets.

**Figure 3:**
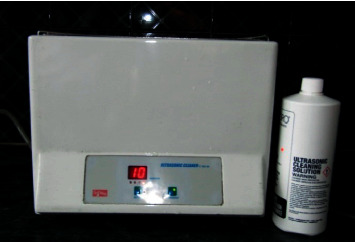
Ultrasonic cleaning of brackets.

Debonding of brackets in Group II, Group III, and Group IV was performed using peeling type forces before recycling as recommended by Zachrisson and Büyükyılmaz [20]. Recycling of brackets in Group II was performed using flaming with the reducing zone of the ﬂame of the gas microtorch (RS Pro, Dubai, UAE) for 5 seconds, then quenched in water at room temperature, and dried in air stream (Figure 1). Group III brackets were subjected to flaming for 5 seconds, quenched in water at room temperature, and dried in air stream as described above followed by sandblasting with 50 *μ*m aluminium oxide abrasive powders using the Bio-Art sandblaster (São Carlos - SP, Brazil). The distance between the bracket base and the handpiece head was fixed at 10 mm [4]. Each bracket was sandblasted for 25 seconds under 5 bar (72.5 psi) line pressure [4] (Figure 2). In Group IV brackets, flaming was performed using the same protocol followed by ultrasonic cleaning using the ultrasonic cleaning solution from Gemoro ultrasonic parts cleaner solution solvent fluid, USA, in an ultrasonic cleaning unit (Confident Dental Equipments Pvt Ltd, Delhi, India) for 10 minutes [4] (Figure 3).

**Figure 4:**
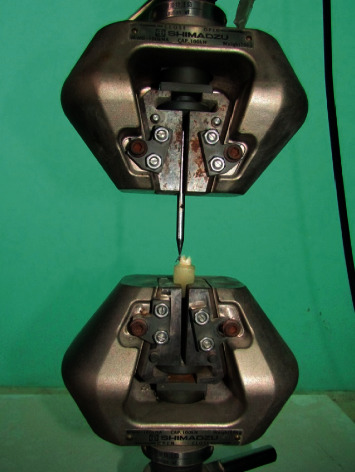
Close view of crosshead of the universal testing machine with sample in situ.

Composite was removed from the tooth surface with sixteen fluted tungsten carbide bur in unidirectional movement [21] with a water cooling system until there was no visible adhesive remaining on the tooth surface [20]. All recycled brackets were bonded to teeth using the standard bonding procedure as described above. All samples were stored in distilled water until final debonding was performed. Final debonding was performed immediately after 24 hours of bonding to standardize shear bond strength in a universal testing machine [11, 12, 15] (AG-IC/100 KN, Shimadzu, Japan) (Figure 4) available at the Nepal Bureau of Standards and Metrology, Balaju, at a crosshead speed of 0.5 mm/min [15]. The force required to dislodge the brackets was measured in Newton, and the shear bond strength (MPa) was calculated by dividing the force values with the bracket base area of 11.6 mm^2^.

(1) $$\begin{array}{c} \text{SBS }\left(\text{MPa}\right)\;=\;\frac{\text{peak load at failure }\left(N\right)}{\text{specimen surface area }{\left(\text{mm}\right)}^{2}}. \end{array}$$

After bond strength testing, all specimens were collected and visually examined using a stereomicroscope (Olympus SZX12; Olympus corp., Tokyo, Japan) at 10X magnification to assess the adhesive remnant index [15, 22] available at Nepal Agricultural Research Council (NARC), Khumaltar. The adhesive remnant index was used to evaluate the amount of resin remaining on the tooth after debonding. At the beginning of the experiment, assessment of intraobserver reliability was done for which the entire procedure was performed by single person and the observation of shear bond strength was also done by the same observer where twenty percentage of samples from each group were randomly selected and subjected to respective methods of recycling. Shear bond strength was recorded using a universal testing machine (*T*_1_). Same procedure was repeated after 2 weeks of the first observation, and shear bond strength was recorded (*T*_2_). The data were processed and analyzed using the Statistical Package for the Social Sciences software, version 21.0 (SPSS Inc. Chicago, Illinois, USA), where descriptive statistics, analysis of variance, and Tukey's post hoc multiple comparison test were used and statistical significance was set at *p* less than 0.05. The Kolmogorov–Smirnov test and the Shapiro–Wilk test were used for test of normality.

## 3. Results

**Figure 5:**
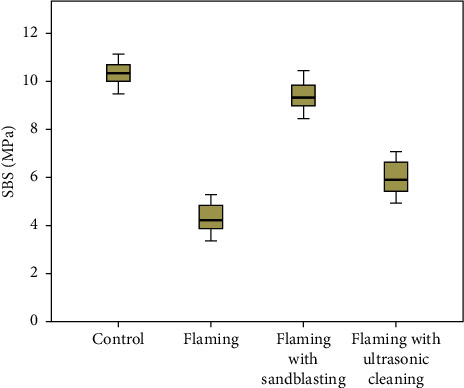
Box plot for mean SBS of different groups.

The intraclass correlation coefficient (ICC) of shear bond strength of brackets selected for the reliability test and subjected to respective methods of recycling at *T*_1_ and *T*_2,_ which showed good intrapersonal reliability of shear bond strength between two measurements (ICC: 0.905) (Appendix 1). Findings of the Kolmogorov–Smirnov test and Shapiro–Wilk test used for test of normality showed that the data were normally distributed in all four groups (Appendix 2). The mean and standard deviation values of shear bond strength obtained from four groups are shown in Table 1. The highest SBS was obtained with the control group (10.35 ± 0.46 MPa), which was followed by the flaming with sandblasting group (9.36 ± 0.55 MPa) and the flaming with ultrasonic cleaning group (5.97 ± 0.66 MPa), and the least SBS was obtained with the flaming only group (4.30 ± 0.55 Mpa). The graphical representation of mean shear strength value by a box plot diagram is shown in Figure 5. The ANOVA test was used to compare the mean values of shear bond strength obtained in each group (Table 2). The test showed that the difference in the mean values of shear bond strength was statistically significant (*p* < 0.001). Tukey's post hoc multiple comparison test (Table 3) was used for intergroup comparisons. All statistical analyses were conducted at a significance level of 0.05. The test showed that the shear bond strength of each group was significantly different from one another. *p* values less than 0.05 in both the ANOVA test and Tukey's post hoc test led to rejection of null hypothesis and acceptance of alternate hypothesis. Hence, there is difference in shear bond strength of stainless steel orthodontic brackets recycled with different methods. Adhesive remnant index scores (by Artun and Bergland [23]) based on the amount of resin left on the tooth after debonding of the four groups are shown in Table 4. The chi-square test was used to compare the ARI values (Table 5) found for each group and that detected statistically significant difference in the adhesive remnant index scores of the 4 groups (*p* < 0.001), i.e., the method of recycling influenced the ARI. Group I and Group III showed predominant scores 0 and 1, Group II showed predominant scores 2 and 3, and the Group IV showed predominant scores 1 and 2.

## 4. Discussion

The goal of reconditioning of orthodontic brackets is to remove the adhesive from the bracket completely without damaging or weakening the delicate base or distorting the dimensions of the bracket slot. The present study compared the shear bond strength of rebonded brackets that were reconditioned by three office reconditioning methods.

Research evaluating the effect of the storage media on bond strength has found that distilled water storage did not adversely affect the bond strength of the teeth stored for up to 6 months [24, 25]. Zachrisson and Büyükyılmaz recommended using peeling type forces, which allow for a recycling process without deformation of bracket during the removal (23). Debonding with peeling force is easily performed by eliminating the peripheral stresses with low force (Oilo) [26]. Hence, peeling type force was used for debonding in this study. Buchman [27] stated that when the stainless steel bracket is subjected to high temperature, chromium carbide precipitate is formed, leading to general weakening of the structure. Accordingly, flaming for 5 seconds was used by Bansal and Bansal [11], Bahnasi et al. [8], and Chetan [4].Hence, in this study, flaming was done for 5 seconds, then quenched in water at room temperature, and dried in air stream.

In this study, the mean shear bond strength of the new brackets was 10.35 ± 0.46 MPa. Flaming with sandblasting showed the highest mean shear bond strength of 9.36 ± 0.55 MPa among the reconditioned methods tested followed by flaming with ultrasonic cleaning, i.e., 5.97 ± 0.66 MPa, and direct flaming, i.e., 4.30 ± 0.55 MPa. This finding is consistent with the study by Chetan [4]. This might be due to obstruction of the mechanical retentive areas with char in flamed brackets, which is partially removed in ultrasonic cleaning and greatly removed in sandblasting. Reynolds gave 5.9 MPa to 7.8 MPa as the optimal range for bond strength required clinically [28]. The results of the present study indicates that the bond strengths of brackets reconditioned by flaming with ultrasonic cleaning and flaming with sandblasting fall under the optimal range for bond strength required clinically. In this study, the mean shear bond strength of brackets recycled by flaming with ultrasonic cleaning is 5.97 ± 0.66 MPa, which falls in the lower limit of the recommended optimal range for bond strength required clinically. Though this finding agrees with that of Chetan [4], it differs from the result of Quick et al. [5] (4.24 ± 2.54 MPa) and Kumar et al. [14] (5.56 ± 0.92 Mpa). The results of this study agree with that of Regan et al. [7]; they compared the initial bond strength and rebond strength of metal brackets and found that the initial bond strength was significantly greater than that of rebond strength of flamed brackets.

Quick et al. [5] reported in their study that flamed, ultrasonically cleaned brackets had significantly lower bond strength than new brackets and indicated that ultrasonically cleaning for 5 minutes was insufficient to dislodge the residue. In a study by Chetan [4], timing for ultrasonic cleaning was increased to 10 minutes. The results of the bond strength tests of that study showed that flamed, ultrasonically cleaned brackets had slightly higher bond strength (6 MPa). In this study, ultrasonic cleaning of flamed brackets was also done for 10 minutes and mean shear bond strength (5.97 Mpa) was reported similar to that in the study by Chetan. This value though falls in the lower limit of the recommended optimal range is still significantly lower bond strength than new brackets. This indicates that either flaming for 5 seconds was insufficient to combust all the composite or that ultrasonic cleaning for 10 minutes was insufficient to dislodge the residue. Based on the study by Kumar et al. [14], flaming followed by ultrasonic cleaning, electropolishing, and silane coupling agent application could be a viable option of recycling brackets to achieve adequate shear bond strength. Quick et al. [5] found that the shear bond strength of flamed followed by sandblasted brackets is not statistically different from that of new brackets. Bansal and Bansal [11] investigated six different reconditioning methods of brackets and found the lowest shear bond strength in the flaming group; however, the values of that study did not correlate with that of other studies reported in the literature. The authors stated, “this difference could be attributed to the type of bracket, adhesive used, and variations in standardization procedures.” Shetty et al. [12] reported in a study that the shear bond strength of brackets recycled by sandblasting with 50-µm aluminum oxide produced a bond strength value of 9.11 ± 4 Mpa, which is slightly less than the bond strength of the present study. This might be due to the difference in pressure used in sandblasting and crosshead speed. In the present study, 5 bar (72.5 psi) pressure was used and crosshead speed was set at 0.5 mm/min, while Shetty et al. used 2.5 bar pressure and crosshead speed was set at 1 mm/min.

Shear bond strength studies are well accepted in Dentistry and Orthodontics in order to have a preliminary test about materials [29]; however, in vitro tests should be confirmed with randomized clinical trials. In fact the results could be different between the two study methodologies [30]. Many variables could alter bond strength values, such as thermocycling [31], enamel contamination [32], or adhesive system used [33]. Therefore, further research is needed in order to confirm the results of the present report.

This in vitro study fails to simulate factors such as intraoral aging of resin composites, PH, and temperature fluctuation based on individual's dietary intake and oral hygiene, complex cyclic loading, microbial attack, and enzymatic degradation. This study used the universal testing machine under a constant crosshead speed of 0.5 mm/minute for bracket removal, which may not correspond to clinical conditions since debonding in vivo occurs at a higher speed. [34].

## 5. Conclusion

Based on the analysis of the data obtained in this study, the following conclusions are made:

- Shear bond strength of the new brackets was significantly higher than that of the recycled brackets.
- Flaming with sandblasting as a method of recycling brackets provided adequate shear bond strength for clinical use. Hence, sandblasting should be considered as a viable, time-saving, and convenient method of chairside recycling.
- Recycling brackets using flaming with ultrasonic cleaning provided shear bond strength falling in the lower limit of the optimum recommended range by Reynolds.
-  Flaming alone led to significantly lower shear bond strength than the recommended range and can be eliminated as a chairside recycling method.

## Figures and Tables

**Table 1:** Shear bond strength mean values (in MPa) of different groups.

Group	*N*	Mean	Std. deviation	Minimum	Maximum	Median
Group I (control)	30	10.35	0.46	9.48	11.12	10.34
Group II (flaming)	30	4.30	0.55	3.36	5.26	4.22
Group III (flaming with sandblasting)	30	9.36	0.55	8.45	10.43	9.31
Group IV (flaming with ultrasonic cleaning)	30	5.97	0.66	4.91	7.07	5.91

**Table 2:** Analysis of variance for comparisons of shear bond strength mean values in different groups.

	Sum of squares	Df	Mean square	F	Sig.
Between groups	723.96	3	241.32	776.13	<0.001
Within groups	36.07	116	0.31	—	—
Total	760.03	119	—	—	—

**Table 3:** Tukey's post hoc multiple comparison test for intergroup comparisons.

(I) group	(J) group	Mean difference (I-J)	Std. Error	Sig.	95% confidence interval	
					Lower bound	Upper bound
Group I (control)	Group II (flaming)	6.046^*∗*^	0.144	<0.001	5.67	6.42
	Group III (flaming with sandblasting)	0.989^*∗*^	0.144	<0.001	0.61	1.36
	Group IV (flaming with ultrasonic cleaning)	4.376^*∗*^	0.144	<0.001	4.00	4.75

Group II (flaming)	Group I (control)	−6.046^*∗*^	0.144	<0.001	−6.42	−5.67
	Group III (flaming with sandblasting)	−5.057^*∗*^	0.144	<0.001	−5.43	−4.68
	Group IV (flaming with ultrasonic cleaning)	−1.670^*∗*^	0.144	<0.001	−2.04	−1.29

Group III (flaming with sandblasting)	Group I (control)	−0.989^*∗*^	0.144	<0.001	−1.36	−0.61
	Group II (flaming)	5.057^*∗*^	0.144	<0.001	4.68	5.43
	Group IV (flaming with ultrasonic cleaning)	3.388^*∗*^	0.144	<0.001	3.01	3.76

Group IV (flaming with ultrasonic cleaning)	Group I (control)	−4.376^*∗*^	0.144	<0.001	−4.75	−4.00
	Group II (flaming)	1.670^*∗*^	0.144	<0.001	1.29	2.04
	Group III (flaming with sandblasting)	−3.388^*∗*^	0.144	<0.001	−3.76	−3.01

^*∗*^ The mean difference is significant at the 0.05 level.

**Table 4:** Adhesive remnant index (ARI) scores for different groups.

Group	ARI				Total
	Score 0	Score 1	Score 2	Score 3	
Group I (control)	8	21	1	0	30
Group II (flaming)	0	0	10	20	30
Group III (flaming with sandblasting)	4	25	1	0	30
Group IV (flaming with ultrasonic cleaning)	1	11	14	4	30

**Table 5:** Chi-square tests for comparisons of ARI values in different groups.

	Value	Df	*p* value
Pearson chi-square	103.401^a^	9	<0.001
Likelihood ratio	119.940	9	<0.001
Linear-by-linear association	1.833	1	0.176
N of valid cases	120	—	—

^a^4 cells (25.0%) have expected count less than 5. The minimum expected count is 3.25.

## Data Availability

The full dataset supporting the conclusion of this article can be obtained upon request to the corresponding author at purna087@gmail.com.

## Abbreviations

ANOVA: Analysis of variance
ARI: Adhesive Remnant Index
Df: Degree of freedom
ICC: Intraclass correlation coefficient
MPa: Mega Pascal
SBS: Shear bond strength

## Supplementary Materials

Supplementary Materials Appendix 1: Table showing intraclass correlation coefficient (ICC) for reliability test. Appendix 2: Table showing test of normality.
