# Supplementary material for: A Comparative Study on the Effect of Different Methods of Recycling Orthodontic Brackets on Shear Bond Strength
Source: Int J Dent. 2021 Jan 21;2021:8844085. doi: 10.1155/2021/8844085 (PMC7843174; doi:10.1155/2021/8844085)
Supplement: Supplementary Materials — Appendix 1: Table showing intraclass correlation coefficient (ICC) for reliability test. Appendix 2: Table showing test of normality. [file 8844085.f1.docx]

**Supplementary information: Additional file 1**

Appendix 1: Table showing Intraclass Correlation Coefficient (ICC) for reliability test.

|  | | | | | | | |
| --- | --- | --- | --- | --- | --- | --- | --- |
|  | Intraclass Correlation | 95% Confidence Interval | | F Test with True Value 0 | | | |
|  |  | Lower Bound | Upper Bound | Value | df1 | df2 | Sig |
| Single Measures | 0.826 | 0.639 | 0.921 | 10.481 | 23 | 23 | <0.001 |
| Average Measures | 0.905 | 0.779 | 0.959 | 10.481 | 23 | 23 | <0.001 |

Appendix 2: Table showing Test of Normality

|  | | | | | | |
| --- | --- | --- | --- | --- | --- | --- |
|  | Kolmogorov-Smirnov^a^ | | | Shapiro-Wilk | | |
|  | Statistic | Df | Sig. | Statistic | Df | Sig. |
| GroupI | 0.087 | 30 | 0.200^*^ | 0.971 | 30 | 0.561 |
| GroupII | 0.114 | 30 | 0.200^*^ | 0.958 | 30 | 0.272 |
| GroupIII | 0.084 | 30 | 0.200^*^ | 0.974 | 30 | 0.644 |
| GroupIV | 0.116 | 30 | 0.200^*^ | 0.949 | 30 | 0.157 |
| *. This is a lower bound of the true significance. | | | | | | |
| a. Lilliefors Significance Correction | | | | | | |
